# Metabolic adverse events of multitarget kinase inhibitors: a systematic review

**DOI:** 10.1007/s12020-023-03362-2

**Published:** 2023-04-17

**Authors:** Elisa Acitelli, Carlo Maiorca, Giorgio Grani, Marianna Maranghi

**Affiliations:** Department of Translational and Precision Medicine, Sapienza University of Rome, Rome, Italy

**Keywords:** Cholesterol, Triglycerides, Dyslipidemia, Hyperglycemia, Hypoglycemia, Tyrosine kinase inhibitors

## Abstract

**Purpose:**

Multitargeted kinase inhibitors (MKIs) are used for the treatment of several cancers. By targeting multiple signaling pathways, MKIs have become cornerstones of the oncologic treatment. Although their use leads to important results in terms of survival, treatment with MKIs can determine important side effects the clinician must be aware of. Among those, arterial hypertension, mucositis and skin lesions are universally reported, while data about metabolic alterations are scarce. In our review, we focused on glucose and lipid alterations in MKI-treated patients.

**Methods:**

We searched for articles, published between January 2012 and December 2022, evaluating the effects on lipid and glucose metabolism of four MKIs (Cabozantinib, Lenvatinib, Sorafenib, and Vandetanib) in adult patients with cancer. We focused on drugs approved for thyroid malignancies, since a worse metabolic control may potentially impact life expectancy, due to their better overall survival rate.

**Results:**

As for glucose metabolism, the majority of the studies reported elevation of glucose levels (prevalence: 1–17%) with different grades of severity, including death. As for cholesterol, 12 studies reported worsening or new-onset hypercholesterolemia (prevalence: 4–40%). Finally, 19 studies reported different grades of hypertriglyceridemia (prevalence: 1–86%), sometimes leading to life-threatening events.

**Conclusions:**

Despite some inherent limitations, our analysis may cast light upon some of the MKIs metabolic disorders that can impact on patients’ health, especially when long-term survival is expected. Future clinical trials should consider routine assessment of glucose and lipid levels, because underdetection and underreporting of alterations can lead to the overlooking of important adverse events.

## Introduction

Multitargeted kinase inhibitors (MKI) are increasingly approved and used for multiple solid and hematological malignant neoplasms and target several molecular pathways involved with cellular growth and de-differentiation, such as vascular endothelial growth factor, fibroblast growth factor, platelet-derived growth factor, and c-Kit pathways. These drugs are collectively referred to as antiangiogenic drugs, because they also interfere with angiogenesis, to differentiate them from more recent molecules, designed to be selective to specific mutations or pathways [1].

Due to their off-target activities and interactions with multiple kinases, several systemic side effects are commonly reported with these drugs (e.g., arterial hypertension, mucositis, and skin lesions) [2, 3].

During randomized clinical trials, not all adverse events were reported. As commonly recognized, data from controlled clinical trials are rarely reproducible in real-life practice, due to their highly controlled setting (optimized to show the effect of the drug), and their carefully selected populations, usually not the same to which the drug is prescribed in clinical practice [4].

Furthermore, drug side effects are heterogeneous by nature, being influenced by age, drug interactions, pharmacokinetics and pharmacodynamics, and developing methods to identify patients at higher risk has proved challenging [5]. Their underdetection and consequent underreporting may thus lead to an underestimate of the impact on patients’ health.

For example, some subtle, biochemical side effects of Lenvatinib (adrenal insufficiency [6–8], or hypocalcemia) were not specifically reported in the SELECT trial [9], nor in the early real-world reports [10–12]. While the metabolic adverse effects of MKIs are known [13, 14], data on their severity and prevalence are scarce. In this review, we focused on the alterations of glucose and lipid metabolism in patients treated with some commonly used MKIs (Cabozantinib, Lenvatinib, Sorafenib, and Vandetanib).

We chose to focus on drugs approved for thyroid malignancies. These patients have a better overall survival rate (53.3% 5-year relative survival rate, even in cases with distant metastases, Table 1), and a worse metabolic control may impact life expectancy.

**Table 1:** 5-Year Relative Survival Rate (95% CI), according to the Surveillance, Epidemiology and End Results (SEER) database (2012–2018, all races, and all ages)

Cancer (with distant metastases)	5-Year relative survival rate % (95% CI)		
	Overall	Males	Females
Thyroid	53.3 (51–55.6)	51.3 (47.7–54.8)	54.8 (51.7–57.8)
Melanoma	31.9 (30.3–33.6)	31.8 (29.8–33.9)	32.2 (29.3–35.1)
Kidney	15.7 (14.9–16.6)	15.9 (14.8–17.0)	15.4 (13.9–16.9)
Adenocarcinoma of the lung	9.5 (9.2–9.9)	7.6 (7.2–8.0)	11.5 (11.0–12.0)
Glioblastoma of the brain	3.8 (1.9–6.7)	1.4 (0.3–4.6)	8.0 (3.7–14.5)
Liver and Intrahepatic Bile Duct	3.1 (2.7–3.6)	2.9 (2.4–3.4)	3.6 (2.8–4.6)

## Methods

The study protocol was registered on PROSPERO (registration number CRD42023387091).

We performed literature research in twelve databases (PubMed, ISI, mRCT, EMBASE, Cochrane, Clinical trial.gov, Scopus, GHL, POPLINE, Google Scholar, VHL and SIGLE).

The research was carried out using the following wording: (cabozantinib OR lenvatinib OR sorafenib OR vandetanib) AND (“metabolic effects” OR “high glucose” OR “low glucose” OR hyperglycemia OR hypoglycemia OR “glucose alterations” OR dyslipidemia OR hyperlipidemia OR hypercholesterolemia OR cholesterol OR “high cholesterol” OR hypertriglyceridemia OR triglycerides OR “high triglycerides” OR “lipid alterations”). Additional articles were searched analyzing the bibliographic references of the selected articles.

We searched for articles evaluating the effects on glucose and lipid metabolism of four MKIs (Cabozantinib, Lenvatinib, Sorafenib and Vandetanib) in adult patients with cancer.

All types of English-language trials and studies evaluating the effect of Cabozantinib, Lenvatinib, Sorafenib and Vandetanib on glucose or lipid metabolism in adult patients were included in our study.

In vitro studies, studies on animals, no full-text articles, case reports and studies on pediatric population were excluded.

**Fig. 1:**
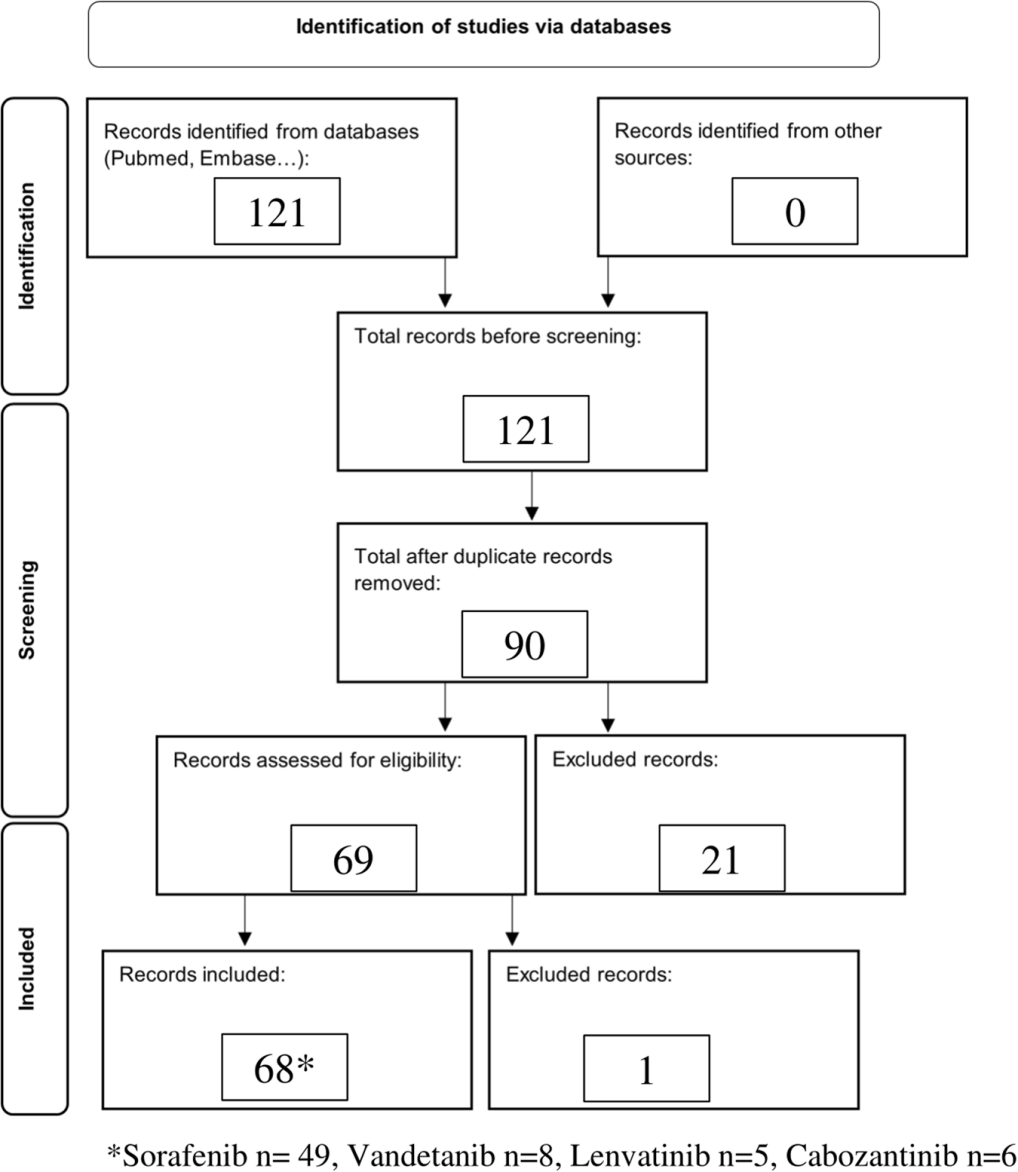
Flow chart of retrieval, inclusion, and exclusion of studies

Studies published before January 2012 and studies involving a population of <10 patients were subsequently excluded as well (see Fig. 1).

The initial research in PubMed, EMBASE, Cochrane, Scopus and POPLINE provided 15, 98, 37, 333, and 203 studies respectively. After title and abstract screening, respectively 12, 5, 19, 82, and 3 were selected. Research through the other databases did not provide results consistent with our inclusion criteria. After duplicates were removed, 90 studies were selected for full-text screening and 69 studies met the inclusion criteria. One study was subsequently excluded because of the plausible confounding effect of one molecule (Trebananib).

The research was independently carried out by two investigators (EA and CM), using the same searching strategy. Only one difference in study selection was assessed, and discussed to reach consensus, with the opinion of a third author (MM).

**Fig. 2:**
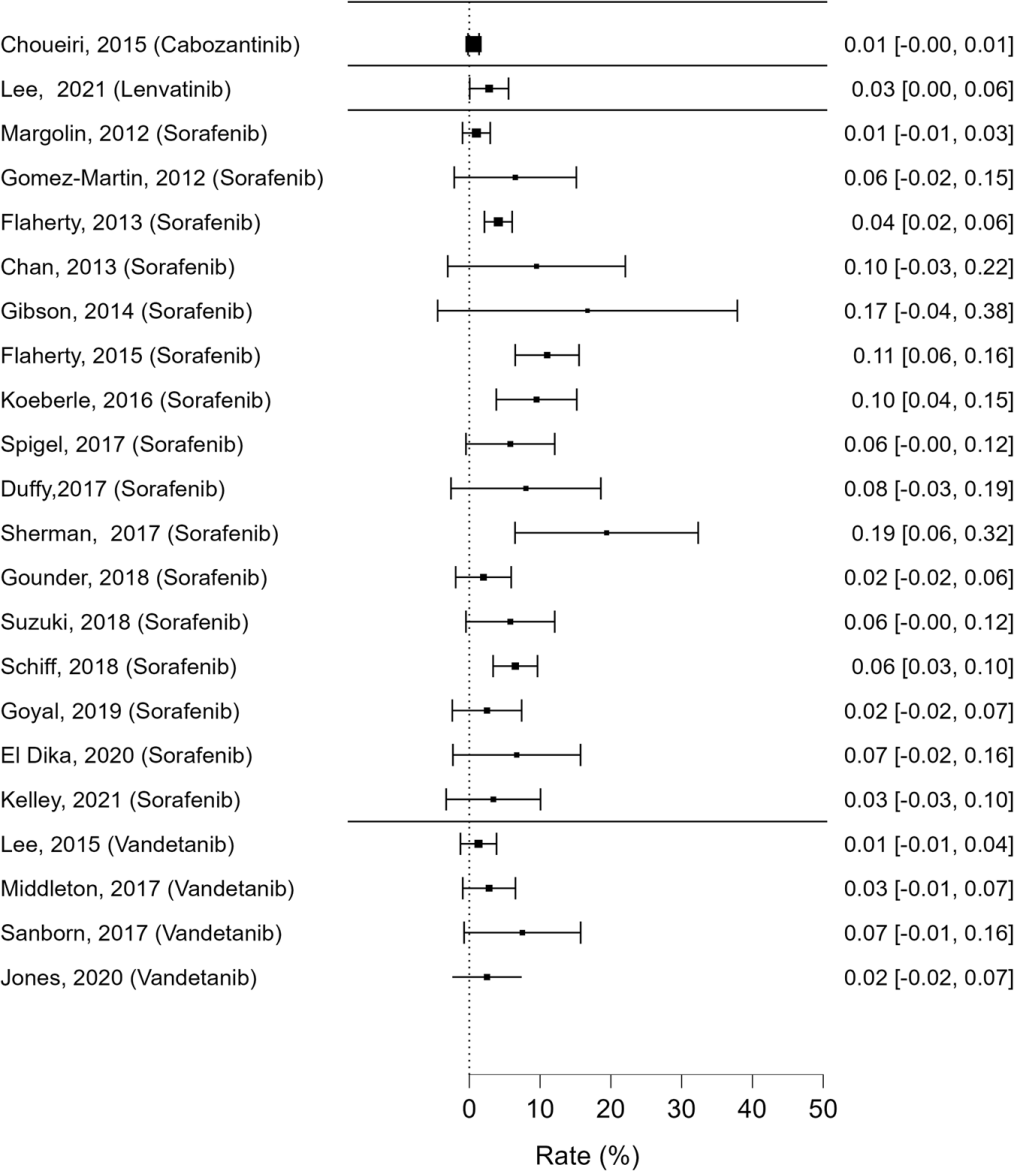
Reported rates of hyperglycemia events (CTCAE grade > 2), along with their 95% confidence intervals

The following data were extracted from each article: type of study, publication year, name of the journal, molecule/s analyzed, recruitment time, population (number of patients), sex, median age, underlying pathology, comorbidities, duration of therapy, daily dose, number of patients undergoing permanent treatment interruption due to adverse events and timing of interruption and effect on glucose, cholesterol and triglyceride metabolism (frequency, timing, grade according to Common Terminology Criteria for Adverse Events [CTCAE]). We chose to report on Table 2 and Fig. 2 only those studies reporting effects on glucose of grade > or = to 3 according to the CTCAE classification.

**Table 2:** Adverse events impacting glucose metabolism reported in the reviewed studies according to CTCAE classification

Author	Molecules	Study design	Patients (n)	Median age (y)	Males (%)	Tumor type	Hyperglycemia (CTCAE grade 3–4), n(%)	CTCAE version
Margolin, 2012 [39]	Sorafenib + Temsirolimus/Tipifarnib	RCT	102	62	55	Melanoma	1 (1)	3
Flaherty, 2013 [40]	Carboplatin + Paclitaxel ± Sorafenib	RCT	823 (*n* Sorafenib = 410)	58	58	Melanoma	17 (4.3)	3
Flaherty, 2015 [41]	Bevacizumab (A) vs Bevacizumab + Temsirolimus (B) vs Bevacizumab + Sorafenib (C) vs Temsirolimus+Sorafenib (D)	RCT	361 (*n* C + D = 181)	na	73	RCC	20 (11)	na
Lee, 2015 [42]	Temozolomide + RT ± Vandetanib	RCT	106 (*n* Vandetanib = 76)	58	58	Glioblastoma	1 (1.4)	3
Choueiri, 2015 [43]	Cabozantinib vs Everolimus	RCT	658 (*n* Cabozantinib= 331)	62	75	RCC	2 (<1)	4
Koeberle, 2016 [44]	Sorafenib ± Everolimus	RCT	105	66	84	HCC	10 (9)	3
Spigel, 2017 [45]	Sorafenib ± Erlotinib	RCT	52	65	35	NSCLC	3 (6)	na
Middleton, 2017 [46]	Gemcitabine ± Vandetanib	RCT	142 (*n* Vandetanib = 72)	67	41	Pancreatic cancer	2 (3)	4.02
Sanborn, 2017 [47]	Etoposide + Platinum ± Vandetanib	RCT	73 (*n* Vandetanib = 40)	64	56	SCLC	3 (7.5)	na
Gounder, 2018 [48]	Sorafenib vs placebo	RCT	87 (*n* Sorafenib = 50)	37	31	Desmoid tumor	1 (2)	4.03
Jones, 2020 [49]	Gemcitabine + Carboplatin ± Vandetanib	RCT	82 (*n* Vandetanib = 40)	73.5	82	Urothelial cell cancer	1 (2)	4
Gomez-Martin, 2012 [50]	Sorafenib + MTOR inhibitors	R	31	54	87	HCC	2 (6)	3
Chan, 2013 [51]	Sorafenib + Everolimus	P	21	53	52	Neuroendocrine tumors	2 (9)	3
Gibson, 2014 [52]	Sorafenib	P	12	69	25	T-Cell lymphoma	2 (16)	na
Sherman, 2017 [53]	Sorafenib + Temsirolimus	P	36	na	53	Thyroid cancer	7 (19)	na
Duffy, 2017 [54]	Sorafenib + TRC105	P	25	60	76	HCC	2 (8)	4
Suzuki, 2018 [55]	Sorafenib	P	52	68	82	HCC	3 (5.8)	4
Schiff, 2018 [56]	Sorafenib + Temsirolimus	P	230	na	na	Glioblastoma	15 (6)	3
Goyal, 2019 [57]	Sorafenib + FOLFOX	P	40	65	85	HCC	1 (3)	4
El Dika, 2020 [58]	Sorafenib + Doxorubicin	P	30	65	87	HCC	2 (7)	3
Kelley, 2021 [59]	Sorafenib + Temsirolimus	P	29	61	86	HCC	1 (4)	4
Lee, 2021[60]	Lenvatinib + Pembrolizumab	P	145	60	78	RCC	4 (3)	4.03

*HCC* hepatocellular carcinoma, *MTOR* mammalian target of rapamycin, *na* not available, *NSCLC* non small cell lung cancer, *P* prospective, *R* retrospective, *RCC* renal cell carcinoma, *RCT* randomized controlled trial, *SCLC* small cell lung cancer

### Metabolic effects of multitargeted kinase inhibitors

Molecularly targeted therapy has become one of the cornerstones of personalized medicine in the oncology field and consists of drugs that specifically interfere with dysregulated signaling pathways in neoplastic cells. Currently, a large spectrum of drugs interfering with cancer cells functions or the tumor microenvironment is being developed. Among those, MKIs target common mechanisms of proliferation, local invasion, metastasis and angiogenesis [15].

Presently, MKIs are widely used for treating several classes of malignant disease, both as a single agent or in combination, and their additional mechanisms of action are still not completely understood [16]. When well tolerated, treatment with a MKI can lead to significant results in terms of overall survival but, occasionally, especially when used in long-term protocol treatments, they can also determine important side effects the clinician must be aware of [3]. Among these effects, glucose and lipids serum levels alterations are not always investigated [17].

As for *glucose metabolism*, many evidences suggest that MKIs can influence glucose levels through different pathways. Most importantly, MKIs belonging to the same class can determine both hyper- or hypoglycemia [18]. Only a small number of reports (3/68) [19–21] described glucose-lowering effects associated with MKIs use. On the other hand, a great number of studies (42/68) reported elevation of serum glucose levels in patients treated with MKIs, while neutral effects were reported in a minority of cases (25/68): 18 studies reported only grade 1 or 2 hyperglycemia [22–38], while the others reported also CTCAE grades 3 or 4 (Table 2). Interestingly, Sorafenib is frequently associated with all CTCAE grades of hyperglycemia, including death, both as a single agent or in combination with other MKIs (Table 2, Fig. 2). Furthermore, Sorafenib is the only MKI, among those included in our review, known to determine hypoglycemic episodes of different severity, including grade 3 or greater, in patients treated for hepatocellular carcinoma or glioma [19–21]. Cabozantinib, Lenvatinib and Vandetanib are mostly associated with mild to moderate high blood glucose (Table 2) and no studies showed evidence of grade 5 hyperglycemia or hypoglycemia associated with their use.

**Fig. 3:**
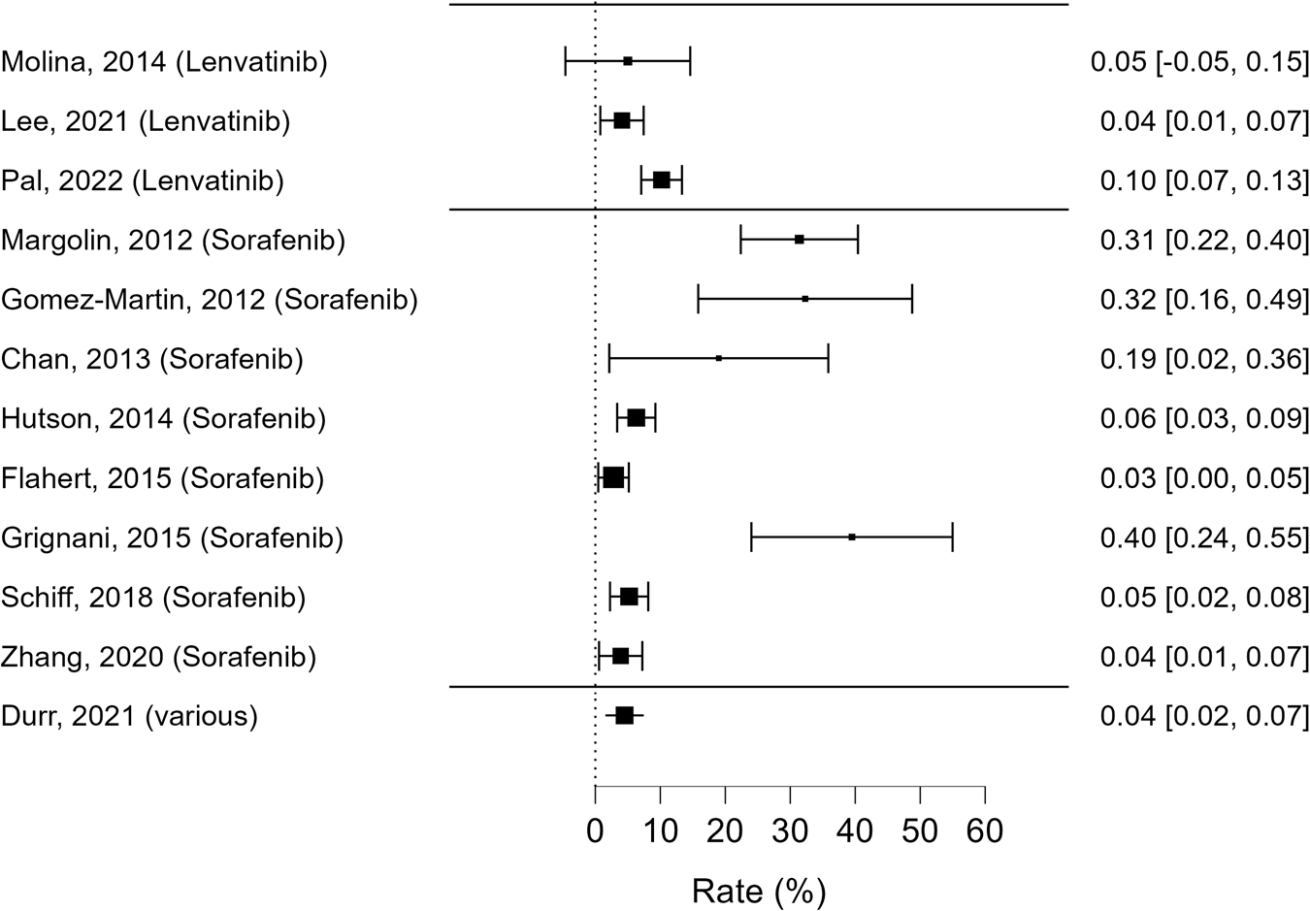
Reported rates of hypercholesterolemia events (all grades), along with their 95% confidence intervals

As regards *cholesterol metabolism*, worsening or new onset of high serum cholesterol levels has been reported in only 12 studies (Table 3, Fig. 3). Most studies (7/12) reported CTCAE grade 3 and 4 hypercholesterolemia, while grade 5 was not reported. Sorafenib, Cabozantinib and Lenvatinib have all been implicated in new onset of different grades of hypercholesterolemia, either when used as a single agent or in combination with other MKIs. Cholesterol metabolism alterations associated with Vandetanib were not described.

**Table 3:** Adverse events impacting cholesterol metabolism reported in the reviewed studies according to CTCAE classification

							Hypercholesterolemia		
Author	Molecules	Study design	Patients (*n*)	Age (y)	Males (%)	Tumor type	CTCAE Grade 1–2 *n* (%)	CTCAE Grade 3–4 *n* (%)	CTCAE version
Margolin, 2012[39]	Sorafenib + Temsirolimus/Tipifarnib	RCT	102	62	55	Melanoma	32 (32)	0	3
Hutson, 2014 [61]	Sorafenib vs Temsirolimus	RCT	512 (*n* Sorafenib = 253)	60	75	RCC	13 (5)	3 (1)	3
Flaherty, 2015 [41]	Bevacizumab (A) vs Bevacizumab + Temsirolimus (B) vs Bevacizumab + Sorafenib (C) vs Temsirolimus + Sorafenib (D)	RCT	361 (n C + D = 181)	na	73	RCC	na	5 (2)	na
Durr, 2021[62]	All TKI vs standard of care	RCT	202	66	53.5	Solid and hematologic tumors	8 (2)	1 (0.2)	4.03
Pal, 2022[63]	Lenvatinib + Everolimus	RCT	343	61	76	RCC	32 (9)	3 (1)	4.03
Gomez-Martin, 2012[50]	Sorafenib + MTOR inhibitors	R	31	54	87	HCC	10 (32)	0	3
Zhang,2020 [64]	Sorafenib	R	127	55	70	RCC	5 (38) total	na	na
Chan, 2013[51]	Sorafenib + Everolimus	P	21	53	52	Neuroendocrine tumors	4 (22)	0	3
Molina, 2014[65]	Lenvatinib + Everolimus	P	20	58	70	RCC	na	1 (5)	na
Grignani, 2015[66]	Sorafenib + Everolimus	P	38	31	61	Osteosarcoma	15 (39)	0	4.03
Schiff, 2018[56]	Sorafenib + Temsirolimus	P	230	na	na	Glioblastoma	na	12 (5)	3
Lee, 2021[60]	Lenvatinib + Pembrolizumab	P	145	60	78	RCC	5 (3)	1 (1)	4.03

*HCC* hepatocellular carcinoma, *MTOR* mammalian target of rapamycin, *na* not available, *P* prospective, *R* retrospective, *RCC* renal cell carcinoma, *RCT* randomized controlled trial, *TKI* tyrosine kinase inhibitors

**Fig. 4:**
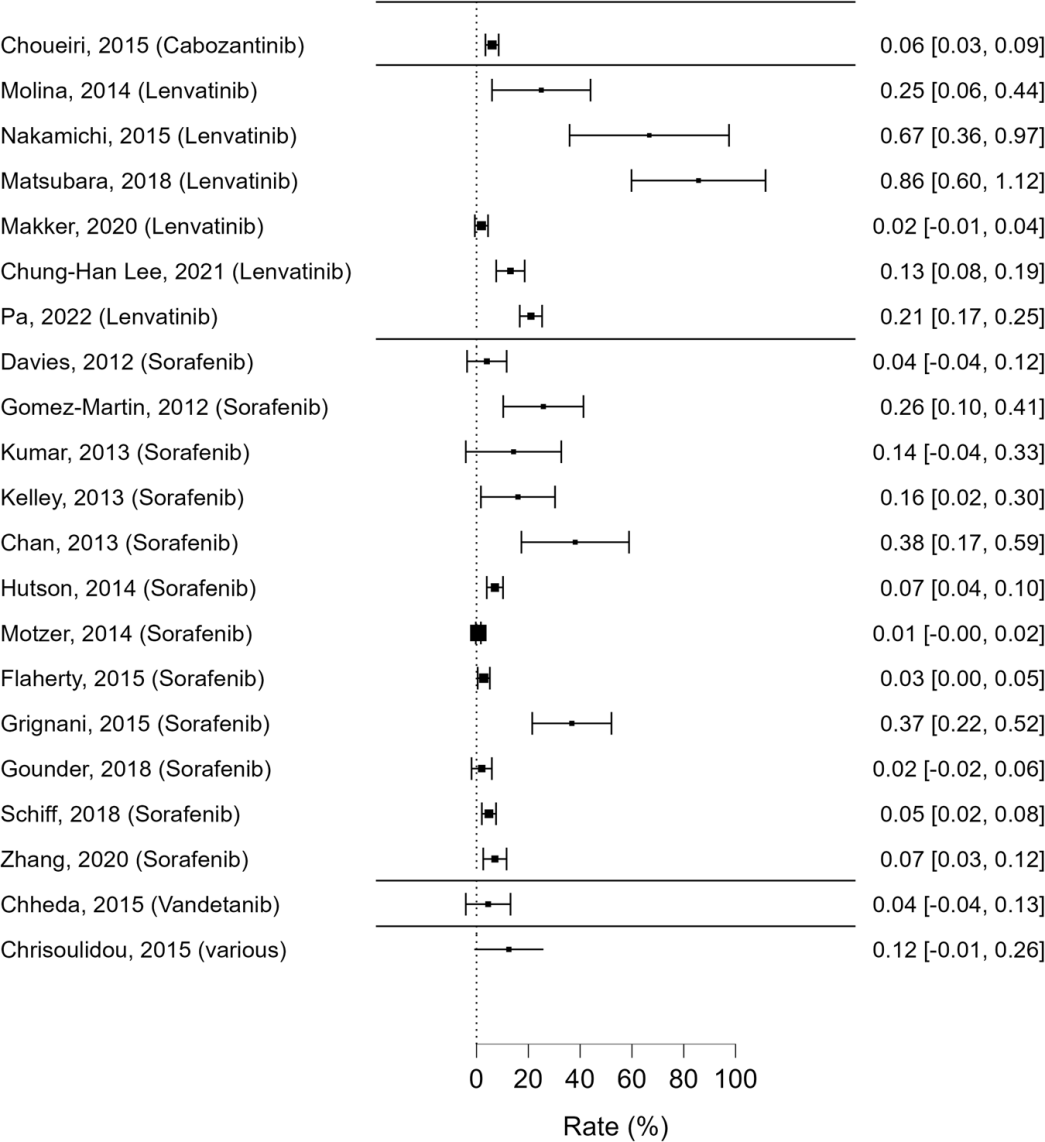
Reported rates of hypertriglyceridemia events (all grades), along with their 95% confidence intervals

Regarding *triglycerides metabolism*, 19 studies reported on the occurrence of hypertriglyceridemia in patients treated with MKIs. Cabozantinib, Lenvatinib, Sorafenib and Vandetanib adversely affect triglycerides metabolism with different degrees of severity, from mild to life-threatening levels (Fig. 4, Table 4).

**Table 4:** Adverse events impacting triglycerides metabolism reported in the reviewed studies according to CTCAE classification

							Hypertriglyceridemia		
Author	Molecules	Study design	Patients (*n*)	Age (y)	Males (%)	Tumor type	CTCAE Grade 1–2 *n* (%)	CTCAE Grade 3–4 *n* (%)	CTCAE version
Motzer, 2014 [67]	Sorafenib vs Dovitinib	RCT	570 (*n* Sorafenib = 286)	62	77	RCC	1 (<1)	1 (<1)	4.03
Hutson, 2014 [61]	Sorafenib vs Temsirolimus	RCT	512 (*n* Sorafenib = 253)	60	75	RCC	17 (6)	1 (<1)	3
Flaherty, 2015 [41]	Bevacizumab (A) vs Bevacizumab + Temsirolimus (B) vs Bevacizumab + Sorafenib (C) vs Temsirolimus + Sorafenib (D)	RCT	361 (*n* C + D = 181)	na	73	RCC	na	5 (2)	na
Choueiri, 2015 [43]	Cabozantinib vs Everolimus	RCT	658 (*n* Cabozantinib = 331)	62	75	RCC	15 (5)	5 (2)	4
Gounder, 2018 [48]	Sorafenib vs placebo	RCT	87 (*n* Sorafenib = 50)	37	31	Desmoid tumors	na	1 (2)	4.03
Pal, 2022 [63]	Lenvatinib + Everolimus	RCT	343	61	76	RCC	36 (10)	36 (10)	4.03
Gomez-Martin, 2012 [50]	Sorafenib + MTOR inhibitors	R	31	54	87	HCC	8 (26)	0	3
Pazaitou-Panayiotou, 2015 [68]	Sorafenib/Sunitinib/Vandetanib	R	24	58	58	Thyroid cancer	3 (12)	0	3
Zhang, 2020 [64]	Sorafenib	R	127	55	70	RCC	9 (69) total	na	na
Davies, 2012 [69]	Sorafenib + Temsirolimus	P	25	51	72	Melanoma	na	1 (4)	3
Kumar, 2013 [70]	Sorafenib + Bortezomib	P	14	65	50	Advanced malignancies	2 (14)	0	3
Kelley, 2013 [24]	Sorafenib + Temsirolimus	P	25	76	60	HCC	4 (16)	0	3
Chan, 2013 [51]	Sorafenib + Everolimus	P	21	53	52	Neuroendocrine tumors	7 (33)	1 (5)	3
Molina, 2014 [65]	Lenvatinib + Everolimus	P	20	58	70	RCC	2 (10)	3 (15)	na
Grignani, 2015 [66]	Sorafenib + Everolimus	P	38	31	61	Osteosarcoma	14 (37)	0	4.03
Chheda, 2015 [27]	Vandetanib + Sirolimus	P	22	52.5	64	Glioblastoma	na	1 (5)	3
Schiff, 2018 [56]	Sorafenib + Temsirolimus	P	230	na	na	Glioblastoma	na	11 (4.8)	3
Makker, 2020 [71]	Lenvatinib + Pembrolizumab	P	108	65	0	Endometrial cancer	2 (1.9)	0	4.03
Lee, 2021 [60]	Lenvatinib + Pembrolizumab	P	145	60	78	RCC	13 (9)	6 (4)	4.03

*HCC* hepatocellular carcinoma, *MTOR* mammalian target of rapamycin, *na* not available, *P* prospective, *R* retrospective, *RCC* renal cell carcinoma, *RCT* randomized controlled trial

### Sources of heterogeneity

#### CTCAE definitions

In the examined literature, toxicities are usually graded according to the National Cancer Institute’s Common Terminology Criteria for Adverse Events (CTCAE) on a 1–5 scale: 1 = mild, 2 = moderate, 3 = severe, 4 = life-threatening with urgent intervention indicated, 5 = death related to adverse events (AEs). Interestingly, the reviewed studies assessed AEs using different CTCAE versions (v3.0, v4.0 and v5.0), that differ mainly in the v5.0 definition of hyperglycemia (Table 5).

**Table 5:** Definitions of “hyperglycemia”, “hypoglycemia”, “cholesterol, serum-high or hypercholesterolemia” and “triglyceride, serum-high or hypertriglyceridemia” according to Common Terminology Criteria for Adverse Events (CTCAE) versions 3.0, 4.0 and 5.0

Adverse event (AE)	CTCAE version	Grade 1	Grade 2	Grade 3	Grade 4	Grade 5
Hyperglycemia	v3.0 and v4.0	>ULN – 160 mg/dL	>160–250 mg/dL	>250–500 mg/dL	>500 mg/dL or acidosis	Death
Hyperglycemia	v5.0	Abnormal glucose above baseline with no medical intervention	Change in daily management from baseline for a diabetic; oral antiglycemic agent initiated; workup for diabetes	Insulin therapy initiated; hospitalization indicated	Life-threatening consequences; urgent intervention indicated	Death
Hypoglycemia	v3.0, v4.0 and v5.0	<LLN – 55 mg/dL	<55 – 40 mg/dL	<40–30 mg/dL	<30 mg/dL	Death
Cholesterol, serum-high	v3.0, v4.0 and v5.0	>ULN - 300 mg/dL	>300 - 400 mg/dL	>400–500 mg/dL	> 500 mg/dl	Death
Triglyceride, serum-high	v3.0	>ULN – 2.5 × ULN	>2.5 – 5.0 × ULN	>5.0 – 10 × ULN	>10 × ULN	Death
Triglyceride, serum-high	v4.0 and v5.0	150 mg/dL–300 mg/dL	>300 mg/dL–500 mg/dL	>500 mg/dL – 1000 mg/dL	>1000 mg/dL	Death

*ULN* upper limit normal, *LLN* lower limit normal, *v* version

#### Gender

Most of the patients included in the studies were males, which might represent a potential bias, since lipid metabolism is differentially regulated in males and females.

#### Underreporting

In a minority of papers included in our analysis, toxicities were reported only when graded ≥ 3 according to CTCAE thus leading to important underreporting of mild to moderate adverse events. Furthermore, many studies did not report any glucose or lipid-related AEs, probably due to the lack of planned evaluations in the design of the study. Consistently, this type of AEs are a very small fraction of those reported to the FDA Adverse Event Reporting System (FAERS) (Table 6).

**Table 6:** Rate of metabolic adverse events reported in FDA Adverse Event Reporting System (FAERS) database, as a fraction of all AEs reported

Drug	Glycemia alterations % (n AE/total AE)	Cholesterol alterations % (n AE/total AE)	Triglycerides alterations % (n AE/total AE)
Bevacizumab	0.82 (716/87188)	0.11 (96/87188)	0.11 (99/87188)
Bortezomib	1.06 (506/47491)	0.07 (34/47491)	0.08 (38/47491)
Cabozantinib	1.2 (39/3250)	0.03 (1/3250)	0.06 (2/3250)
Carboplatin + Paclitaxel	2,27 (1/44)	–	–
Doxorubicin	0.74 (329/44708)	0.04 (21/44708)	1.19 (88/44708)
Erlotinib	1.01 (147/14469)	0.05 (7/14469)	0.04 (6/14469)
Etoposide + Cisplatin	6.67 (1/15)	–	–
Everolimus	3.61 (1601/44302)	1.38 (613/44302)	0.85 (378/44302)
Gemcitabine + Carboplatin	18.18 (2/11)	–	–
Gemcitabine	0.87 (208/23828)	0.02 (5/23828)	0.04 (10/23828)
Lenvatinib	1.21 (204/16899)	0.19 (33/16899)	0.11 (19/16899)
Pembrolizumab	1.02 (450/43812)	0.05 (23/42812)	0.04 (20/43812)
Sirolimus	1.12 (166/14734)	0.94 (139/14734)	1.28 (189/14734))
Sorafenib	1.95 (392/20133)	0.15 (30/20133)	0.11 (23//20133)
Sunitinib	2.05 (30/1463)	0.48 (7/1463)	0.2 (3/1463)
Temozolomide	1.45 (290/19976)	0.12 (25/19976)	0.08 (17/19976)
Temsirolimus	2.95 (125/4237)	1.39 (59/4237)	2.05 (87/4237)
Tipifarnib	1.49 (1/67)	–	–
Vandetanib	1.05 (16/1512)	1.52 (23/1512)	1.39 (21/1512)

Metabolic AEs of the same class but with different nomenclatures (e.g., “high glucose”, “hyperglycemia”) were summed up

### Limitations of the review

This systematic review had some limitations. The examined literature reported studies of different designs (randomized clinical trials, prospective studies and retrospective studies) conducted by different research groups in different countries, so potential biases could not be avoided. In many reports, patients were treated with a combination of drugs (MKI plus at least another antineoplastic agent) so that linking the occurrence of specific AEs to a single molecule was not possible (Table 6). Furthermore, some studies evaluated toxicities of severe grades, thus providing only a partial picture of the phenomena described.

Moreover, information about different lipoprotein lipids concentrations (i.e., HDL cholesterol, LDL cholesterol) are lacking in the selected articles, limiting the understanding of the potential atherogenic risk linked to these metabolic disturbances.

In our opinion, clinicians should be familiar with metabolic disorders that MKI-treated patients could develop, including dysglycemia and dyslipidemia. It is important to periodically evaluate glucose and lipid levels in order to recognize and control these adverse events.

In summary, further investigation is necessary for a more comprehensive understanding of the adverse metabolic profile of MKIs. Underdetection and consequent underreporting of potential alterations can lead to the overlooking of important adverse events, especially in the context of MKI- treated patients with a longer overall survival. Future clinical trials should include in their protocol design routine assessment of glucose and lipid profile in order to allow a better understanding of the prevalence of these alterations, identifying subgroups at risk, and possibly paving the way for discovering new molecular mechanisms responsible for these adverse effects.
